# Occurrence and Diversity of Fungi and Their Mycotoxin Production in Common Edible and Medicinal Substances from China

**DOI:** 10.3390/jof11030212

**Published:** 2025-03-10

**Authors:** Ling Chen, Junhui Wu, Shuhong Zhang, Xinqi Liu, Meiping Zhao, Weipeng Guo, Jumei Zhang, Wei Chen, Zhenjie Liu, Meiqing Deng, Qingping Wu

**Affiliations:** 1 College of Food Science, South China Agricultural University, Guangzhou 510640, China; angusy0662@sina.com; 2 National Health Commission Science and Technology Innovation Platform for Nutrition and Safety of Microbial Food, Guangdong Provincial Key Laboratory of Microbial Safety and Health, State Key Laboratory of Applied Microbiology Southern China, Guangdong Detection Center of Microbiology, Institute of Microbiology, Guangdong Academy of Sciences, Guangzhou 510070, China; wujunhui@gdim.cn (J.W.); zhangshuhong@gdim.cn (S.Z.); liuxinqi@gdim.cn (X.L.); zhaomeiping@gdim.cn (M.Z.); guowp@gdim.cn (W.G.); zhangjm926@126.com (J.Z.); chenwei@gdim.cn (W.C.); liuzj@gdim.cn (Z.L.); dengmeiqing@gdim.cn (M.D.)

**Keywords:** edible and medicinal substances, fungal contamination, mycotoxigenic fungi, mycotoxin, food safety

## Abstract

Edible and medicinal substances can be contaminated by fungi during harvesting, processing, and storage, leading to mycotoxin production and quality deterioration. The distribution of mycotoxigenic fungi in edible and medicinal substances was investigated in this study. Fungi and mycotoxins were detected in 163 commercially available edible and medicinal substances using standard microbiological techniques and high-performance liquid chromatography. A total of 92.0% of samples contained fungi (0.5–5.3 lg colony-forming units (CFU)·g^−1^); 208 fungal strains belonging to 16 genera were identified, predominantly *Aspergillus* and *Penicillium. Aspergillus* section *Nigri* (30.3%) produced fumonisin B_2_, which was distributed mainly in radix and rhizome samples. Thirteen samples had mycotoxins, of which ochratoxin A was the most common, followed by aflatoxins and zearalenone (ZEN). One *Nelumbinis* semen sample contained 10.75 μg·kg^−1^ AFB_1_, and one *Raisin tree* semen sample contained 484.30 μg·kg^−1^ ZEN, which exceeded regulatory limits in Europe and China. These findings highlight the potential risks associated with fungal contamination and mycotoxins in edible and medicinal substances. Enhanced quality control measures are essential to reduce contamination during harvesting, processing, and storage. Expanded mycotoxin screening, improved preservation techniques, and stricter regulatory standards need to be implemented to ensure consumer safety.

## 1. Introduction

Edible and medicinal substances are essential for healthcare globally and are traditionally used for disease prevention and treatment [1,2,3]. Approximately 80% of the global population depends on herbal remedies as their primary healthcare solution, and the global herbal supplement market will reach USD 560 billion by 2024 [4]. At present, 106 kinds of medicinal materials have been incorporated into the list of edible and medicinal substances in China [5,6]. Edible and medicinal substances such as *Ganoderma,* with various diverse secondary metabolites (e.g., polysaccharides and triterpenoids), are widely utilized for their immunomodulatory, antioxidant, and antitumor properties [7]. Based on the World Health Organization’s recognition of the value of traditional Chinese medicine in fighting the coronavirus disease (COVID-19) epidemic, edible and medicinal substances are favored globally. However, microbial contamination of edible and medicinal substances has become increasingly prevalent, especially with respect to the presence of mycotoxigenic fungi and mycotoxins, which directly impact the quality, stability, and safety of these preparations [8,9]. For instance, highly nutritious edible and medicinal substances such as *Nelumbinis* semen (lotus seed), *Codonopsis* radix (*Codonopsis* tangshen), *Scutellariae* radix (Baikal skullcap root), and *Angelicae sinensis* radix (Danggui) are easily contaminated by filamentous fungi because of factors such as poor storage conditions, improper harvest and drying practices, and the use of contaminated soil and water [10,11]. In addition, fluctuations in temperature and humidity during storage and transportation can further promote fungal growth, leading to potential mycotoxin biosynthesis. To ensure the safety and quality of edible and medicinal substances, regulatory agencies have established threshold limits for fungal contamination. For example, the European Pharmacopoeia recommends that the total fungal load in herbal materials should not exceed 10^4^ colony-forming units (CFU)/g [12].

*Aspergillus*, *Penicillium*, *Fusarium*, and *Alternaria* are the most predominant genera in edible and medicinal substances, with *Aspergillus* being the most frequently isolated in previous studies [13,14,15,16,17,18,19]. The prevalence of these genera is concerning owing to their ability to produce mycotoxins. For instance, *A. flavus* is among the most important producers of aflatoxins, whereas *A. niger* produces ochratoxin and fumonisin [20]. The presence of these mycotoxin-producing fungi in edible and medicinal substances poses a considerable health risk to humans and animals and has detrimental effects on the quality and safety of food products.

Mycotoxins are secondary metabolites that can cause acute and chronic toxic effects, including carcinogenicity, mutagenicity, and teratogenicity [21,22,23]. Mycotoxins are stable molecules that are difficult to remove or eradicate during processing and most likely remain in the final product. Humans are exposed to mycotoxins by various means, including consumption of contaminated food and beverages, inhalation of airborne mold spores, and dermal contact with surfaces contaminated with mycotoxin-producing fungi [24,25]. In addition, people may ingest products from livestock that consume feed contaminated with mycotoxins [26]. Long-term consumption of edible and medicinal substances contaminated with mycotoxins inevitably increases the occurrence of adverse events and harms human health. Understanding the prevalence and distribution of these fungal species is essential for formulating strategies to prevent and manage the fungal contamination of edible and medicinal substances.

Considering the growing popularity of high-quality edible and medicinal substances and inherent risks posed by fungal contamination, investigating the occurrence and levels of mycotoxigenic fungi and mycotoxins in different types of edible and medicinal substances in specific production areas is crucial. Therefore, this study aimed to: (1) ascertain fungal and mycotoxin levels within edible and medicinal substances, (2) determine the predominant genera using polyphasic taxonomy and analyze the distribution of fungal species, and (3) verify the potential for mycotoxin production.

## 2. Materials and Methods

### 2.1. Edible and Medicinal Substances

For this study, 163 samples from the most used edible and medicinal substances, encompassing 40 distinct varieties divided into six types (animal, edible fungi, flos et folium, fructus, radix et rhizoma, and semen), were selected from specific production regions in China (Table 1). All samples were commercially available products purchased from large Chinese herbal medicine wholesale markets. Each sample was placed in a sterile sampling bag and labeled with a unique identifier corresponding to its origin, variety, and collection date. All samples were transported to the laboratory and identified according to the first part of the Chinese Pharmacopoeia (2020) [27].

### 2.2. Fungal Isolation and Enumeration

The total combined yeast and mold count (TYMC) was determined using the plate-count method, with at least two counts for each medium; counts were averaged. Samples were prepared by homogenizing 25 g of the test sample with 225 mL of a buffered sodium chloride–peptone solution (pH 7.0 ± 0.2), in accordance with the microbiological examination methods specified in the fourth part of the Chinese Pharmacopoeia [27]. Four sequential dilutions were performed for each sample, with dilution factors of 1:10, 1:100, 1:1000, and 1:10,000. Serial decimal dilutions (1 mL each) were inoculated onto a 9-cm diameter Petri dish. Subsequently, 15–20 mL of Sabouraud dextrose agar (SDA) (Guangdong Huankai Microbial Sci & Tech. Co. Ltd., Guangzhou, China) at approximately 45 °C was poured into the dishes. All plates were subsequently incubated at 25 °C for 5–7 d. After incubation, the CFUs were enumerated. The fungal load level was expressed as log TYMC and calculated using Equation (1):

log TYMC = log_10_ (*N* × *D*) (1)

where *N* represents the number of colony-forming units (CFU) per plate and *D* denotes the dilution factor. When the TYMC was <10 (below the detection limit), log TYMC was recorded as 0.5 [28].

### 2.3. Morphological Identification

Three-point inoculations of the isolates were conducted to examine their macromorphology on malt extract agar (MEA) and Czapek yeast extract agar (CYA) (Guangdong Huankai Microbial Sci & Tech. Co. Ltd., Guangzhou, China) following incubation at 25 °C in the dark for 3–7 d [29,30,31]. Colony texture, obverse and reverse colony colors, degree of sporulation, exudates, and pigments were recorded [20]. The microscopic morphology of the fungi was examined using a biological microscope (CX21FS1C; Olympus Ltd., Tokyo, Japan) accompanied by lactophenol cotton blue staining [32]. One to two drops of lactophenol cotton blue stain (Qingdao Hope Bio-Technolongy Co. Ltd., Qingdao, China) were placed on clean microscope slides along with a small amount of spore bearing hyphae from the fungal colony. The hyphae were carefully dispersed, and a cover slip was gently placed over the sample to ensure that no air bubbles were trapped. The sample was then observed under a microscope, starting with a low-power (20×) objective and switching to a high-power (40×) objective if necessary.

### 2.4. Molecular Identification

Fungal DNA was extracted from pure isolates using genomic DNA kits (Omega BioTek Inc., Norcross, GA, USA). The quality and concentration of the extracted DNA were assessed using a NanoDrop 2000 spectrophotometer (Thermo Fisher Scientific, Waltham, MA, USA). Polymerase chain reaction (PCR) assays were performed using the internally transcribed spacer (ITS) region and *β-tubulin* gene. The ITS region was amplified using primers ITS1 (5′-TCC GTA GGT GAA CCT GCG G-3′) and ITS4 (5′-TCC TCC GCT TAT TGA TAT GC-3′); the *BenA* gene encoding *β-tubulin* was amplified using primers Bt2a (5′-GGT AAC CAA ATC GGT GCT GCT TTC-3′) and Bt2b (5′-ACC CTC AGT GTA GTG ACC CTT GGC-3′). The PCR protocols are described by Visagie et al. [33]. The sequences were compared with known fungal sequences using the Basic Local Alignment Search Tool (https://blast.ncbi.nlm.nih.gov/Blast.cgi, accessed on 1 December 2024) to determine the closest matches and confirm the identities of the isolated fungi. Following identification, a phylogenetic analysis was conducted to further elucidate the evolutionary relationships among the different fungal species.

### 2.5. Mycotoxin-Producing Abilities of Aspergillus and Penicillium Strains

One hundred and seventeen strains, comprising 90 *Aspergillus* and 27 *Penicillium* strains, were inoculated into CYA using the method described by Silva et al. [34]. Three small pieces of mycelia (each 9 mm in diameter) were excised from the central portion of the colony using a sterile pipette tip (9 mm caliber) to ensure precision and consistency in the sample size. Mycotoxins were then extracted using 1 mL of 70% methanol in an ultrasonic bath for 30 min, followed by centrifugation at 10,000× *g* for 10 min. Subsequently, the supernatant was transferred to a clean 15 mL centrifuge tube and diluted with ultrapure water to a final volume of 3 mL. The liquid was purified using a Poly-Sery HLB Pro SPE Cartridge (ANPEL Laboratory Technologies Inc., Shanghai, China). Mycotoxins were qualitatively detected by ultra-performance liquid chromatography coupled with tandem mass spectrometry (UPLC–MS/MS) using an LC20 UPLC system paired with a 5500+ mass spectrometer (AB SCIEX, Framingham, MA, USA).

UPLC analysis was performed using a Waters CORTECS C18 column (2.1 × 100 mm, 1.6 μm; Waters Corp., Milford, MA, USA). The column temperature was set at 40 °C. The elution gradient was: 0 min, 95% B; 5 min, 10% B; maintained at 10% B to 8 min; 8.1 min, reverted to 95% B; and held at 95% B to 10 min. The flow rate was set to 0.3 μL·min^−1^. For the analysis of aflatoxins B_1_, B_2_, G_1_, and G_2_, ochratoxin A (OTA), fumonisin B_1_, B_2_, B_3_, and zearalenone (ZEN), the mobile phase was composed of acetonitrile (phase A) and 0.1% acetic acid (phase B). In the case of patulin (PAT), the mobile phase was water (phase A) and acetonitrile (phase B). For citrinin (CTN), the mobile phase comprised acetonitrile (phase A) and 5 mM ammonium acetate (phase B).

Mass analysis was performed using electrospray ionization (ESI) in the positive and negative ion modes, and detection conditions were set as: curtain gas (CUR), 25 psi; GS1, 60 psi; GS2, 60 psi; ion spray voltage (IS), 5500 V (positive mode) and −4500 V (negative mode); and source temperature, 550 °C. The optimal multiple reaction monitoring transitions, dwell times, declustering potentials, and collision energies are summarized in Table S1.

### 2.6. Mycotoxin Determination in Edible and Medicinal Substances

Five grams of each sample were homogenized with 50 mL of a 70% methanol solution, and the resulting mixture was sonicated for 30 min. Following centrifugation at 6000 rpm for 10 min, 10 mL of the supernatant was diluted with 20 mL of ultrapure water, and 3 mL of the diluted liquid was purified using a Poly-Sery HLB Pro SPE Cartridge. The eluate was dried using nitrogen at 50 °C, and the residue was subsequently dissolved in 1 mL of 50% acetonitrile. After vortex-mixing for 30 s, the solution was passed through a 0.22 μm microporous membrane and subjected to qualitative analysis with UPLC–MS/MS. Furthermore, positive samples were confirmed by high-performance liquid chromatography combined with fluorescence detection (HPLC–FLD; LC-20AT with RF-20A fluorescence detector; Shimadzu, Kyoto, Japan) to accurately detect mycotoxin concentration in accordance with the National Food Safety Standards of China methods [35,36,37]. HPLC separation was performed using an Eclipse Plus C18 column (150 × 4.6 mm, 5.0 μm; Agilent, Santa Clara, CA, USA).

Aflatoxin B_1_, B_2_, G_1_, and G_2_ were analyzed at a column temperature of 40 °C and constant flow rate of 1 mL·min^−1^. The mobile phase consisted of water (A) and acetonitrile: methanol (50:50) (B). The gradient elution program was 68% A and 32% B. Excitation and emission wavelengths were set to 360 and 440 nm, respectively, for fluorescence detection. The aflatoxins were determined using a post-column photochemical derivative reactor. For OTA detection, the column temperature was 35 °C, and the mobile phase was acetonitrile–water–acetic acid (96:102:2) at a flow rate of 1.0 mL·min^−1^. For fluorescence detection, the excitation and emission wavelengths were 333 and 460 nm, respectively. ZEN was detected at a column temperature of 25 °C. The mobile phase was acetonitrile–water–methanol (46:46:8) at a flow rate of 1.0 mL·min^−1^. For fluorescence detection, the excitation and emission wavelengths were set to 274 and 440 nm, respectively.

Mycotoxins were quantified by comparing their peak areas with calibration curves obtained using standard solutions. All standard curves exhibited excellent linearity, with correlation coefficients (R²) ranging from 0.9992 to 0.9999. The limits of detection (LOD) and quantification (LOQ) were 0.03–5 and 0.1–17 μg·kg^−1^, respectively (Table S2). In the absence of certified reference materials, the accuracy of the method was assessed through recovery studies by spiking known concentrations of analytes into blank matrices [38,39]. Recovery experiments were conducted at three spiking levels in six different blank matrices. The recovery rates for target mycotoxin ranged from 79.9% to 106.7%, accompanied by relative standard deviations (RSDs) below 5.92% (Table S3).

### 2.7. Statistical Analysis

IBM SPSS (v26) was used for descriptive data analysis and to plot histograms. For inferential statistics, one-way analysis of variance was conducted to determine significant differences among the groups. Gene sequences were aligned, and an evolutionary tree was constructed with the neighbor-joining method using MEGA 7.0.26 [40] and ChiPlot (https://www.chiplot.online/, accessed on 7 February 2024).

## 3. Results and Discussion

### 3.1. Fungal Contamination in Edible and Medicinal Substances

**Figure 1:**
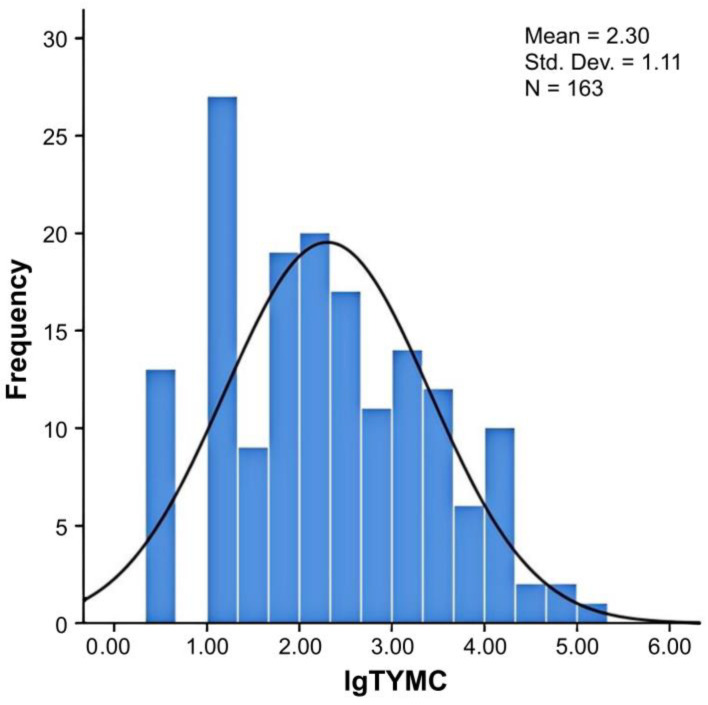
Histogram of log TYMC of edible and medicinal substances. Log TYMC: log total combined yeast and mold count.

Approximately 92.0% (150 of 163) of samples tested positive for fungal contamination. Fungal load per sample was 0.5–5.28 log CFU·g^−1^. The value of log TYMC for edible and medicinal substances was skewed (Figure 1), and only 7.9% (13/163) of the samples exceeded the contamination limit (10^4^ CFU·g^−1^ = 4 lgCFU·g^−1^) recommended by the European Pharmacopoeia [12]. These samples originated from nine edible and medicinal substances: *Acanthopanacis senticosi* radix et rhizoma *seu caulis* (*Acanthopanacis senticosi*), *Cuscutae* semen, *Euryales* semen, *Flammulina velutipes*, *Glycyrrhizae* radix et rhizoma (licorice), *Leonuri herba*, *Mori folium*, *Polygonati rhizoma*, and *Puerariae lobatae* radix (kudzu vine).

Elevated fungal counts may suggest processing failure or recontamination during transportation, storage, or marketing, likely due to increased moisture content. A previous study indicated that the log TYMC ranged from 1 to 7 in Chinese herbal medicines from Shanghai and Beijing [41,42]. Recent studies have focused on fungal levels within a single or limited types of edible or medicinal substrates [43,44,45], often lacking a comprehensive analysis across various herbal medicines or substrates. This may hinder a holistic understanding of the variability and significance of log TYMC levels in Chinese herbal medicines.

Based on the plant parts used, samples were classified into six types: animal, edible fungi, flos et folium, fructus, radix et rhizoma, and semen. The detectable log TYMC values differed by type (Table 2). The log TYMC for radix et rhizoma ranged from 0.5 to 4.80, indicating that the fungal contamination of radix et rhizoma varied greatly, and the quality of the samples was unstable. The roots and rhizomes of medicinal plants are prone to mechanical damage during harvesting, which exposes the tissues and provides conditions for microbial invasion and proliferation [46]. This variability emphasizes the need for stricter quality control measures to ensure the safety and stability of these products.

The higher microbial loads in flos et folium and edible fungi may be attributed to their structural characteristics and moisture content [47], which can create favorable conditions for fungal growth. In contrast, the relatively low microbial loads in animal-based products and fructus may be due to their low moisture content or natural antimicrobial properties [48,49].

The effect of processing methods on microbial contamination is evident. Directly cut and sun-dried slices exhibited higher microbial loads, likely because these methods did not effectively reduce the microbial populations [50]. In contrast, heat processing methods such as boiling, roasting, and steaming substantially reduced microbial contamination, as observed in products such as *Asini corii colla* and *Mume* fructus. This highlights the importance of adopting appropriate processing techniques to minimize the microbial risks in edible and medicinal substances.

### 3.2. Fungal Genera and Species Diversity

A total of 208 strains (196 mold and 12 yeast strains) were isolated from edible and medicinal substances. Preliminary morphological identification indicated the presence of 90 strains of *Aspergillus*; 27 of *Penicillium*; 24 of *Mucor*; 23 of *Rhizopus*; 11 of *Trichoderma*; four of *Alternaria*; four of *Chaetomium*; three of *Talaromyces*; two of *Epicoccum*; two of *Cladosporium*; one each of *Aplosporella*, *Byssochlamys*, *Curvularia*, *Dichotomopilus*, *Neurospora*, and *Periconia*; and 12 unclassified yeasts. Among the isolates, the *Aspergillus* genus was the most prevalent (43.3%), followed by *Penicillium* (13.0%). Silva et al. [34] found that *Aspergillus* was predominant in 30 yerba mates. Yu [51] analyzed the fungal diversity on the surfaces of six herbal materials using high-throughput sequencing and observed the prevalence of *Aspergillus*, *Rhizopus*, and *Penicillium* over other genera. *Aspergillus* and *Penicillium* are common storage fungi used for various edible and medicinal substances, which was consistent with our research [18,52]. Although Wei et al. [18] observed a certain proportion of *Fusarium* (5.17%) in herbal medicines using high-throughput sequencing, traditional culture methods failed to obtain *Fusarium* isolates from 240 samples. A similar response was observed in the present study; we did not isolate *Fusarium* (generally classified as field fungi). This result implies that field fungi can be removed or reduced following processing of edible plant materials, such as washing, drying, or fumigation. *Fusarium* spp. do not usually develop under low humidity, which explains their predominant occurrence in field environments [53]. The low *Fusarium* contents of edible and medicinal substances make them difficult to isolate and identify.

**Figure 2:**
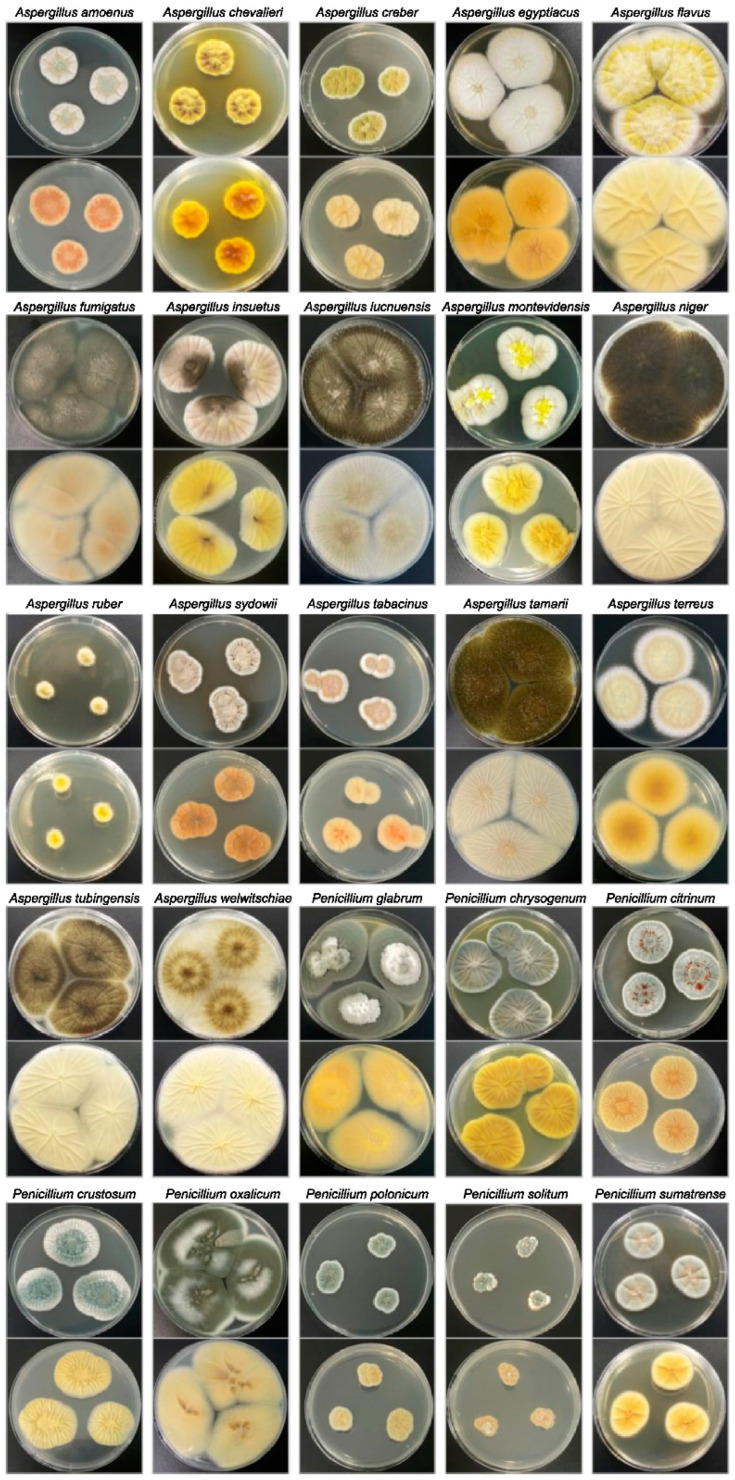
Colony morphology (top and reverse) of *Aspergillus* and *Penicillium* species isolated in this study after 5–10 d of incubation at 25 °C in the dark on Czapek Yeast Extract Agar (CYA).

**Figure 3:**
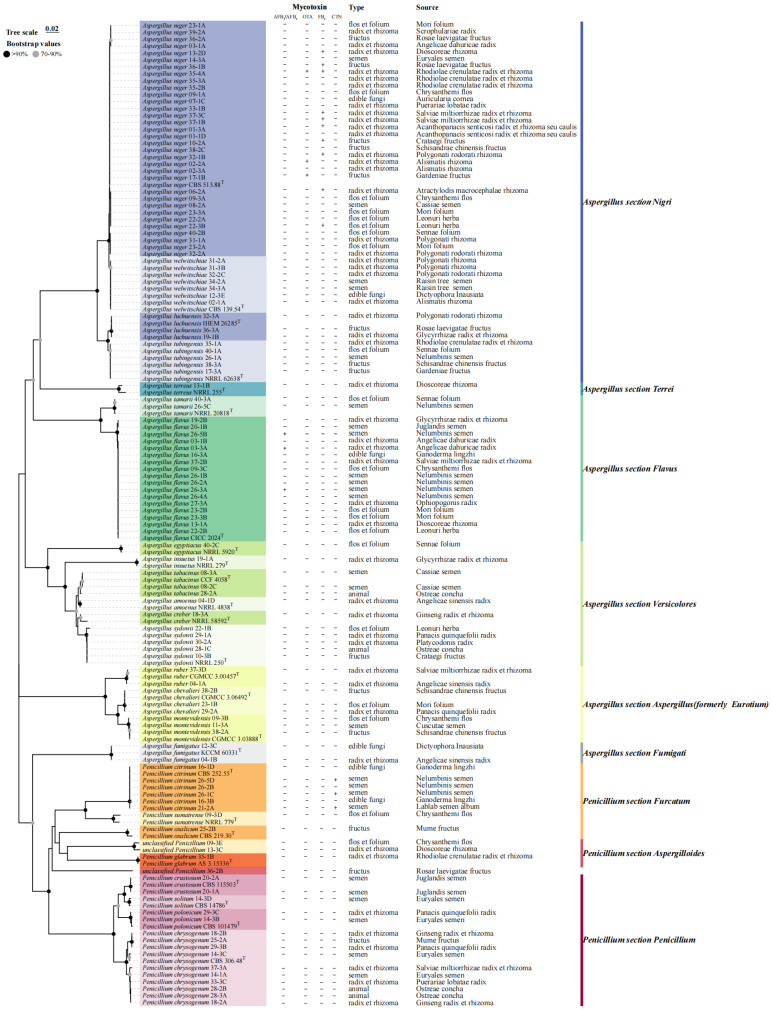
Neighbor-joining tree based on sequence data of internally transcribed spacer (ITS) and β-tubulin from 117 isolates (*Aspergillus* and *Penicillium*) using MEGA 7.0.26. Bootstrap values are shown in the nodes according to 1000 replications. “T” type strain; AF, aflatoxin; OTA, ochratoxin A; FB, fumonisin; CTN, citrinin.

Molecular identification by genetic characterization was used to accurately assign 90 *Aspergillus* and 27 *Penicillium* strains to the species. The colony morphologies of the primary strains (identified at the species level) are shown in Figure 2. Figure 3 shows the phylogenetic tree of the sequences compared with the standard and type strain reference sequences available in GenBank. The 90 *Aspergillus* isolates were divided into six distinct sections: *Nigri*, *Terrei*, *Flavus*, *Versicolores*, *Aspergillus* (formerly *Eurotium*), and *Fumigati*. *Aspergillus* section *Nigri* was the most isolate-rich section, with 48 isolates, including 33 of *A. niger*, seven *A. welwitschiae*, five *A. tubingensis*, and three *A. luchuensis*.

*Aspergillus niger* was the dominant species among the *Aspergillus* isolates, followed by *A. flavus; A. welwitschiae* from the *A. niger* taxon is present in dried fruits, grapes, coffee beans, cocoa, onions, and in rhizosphere soil [15]. In agriculture, *A. welwitschiae* in sisal substances has been implicated as the causative agent of maize ear rot and bole rot disease [54,55]. These infections can lead to considerable crop losses and economic burden for farmers. Furthermore, *A. welwitschiae* is a pathogen in human infections, particularly otomycosis (ear canal infections). This species is often resistant to nystatin, posing challenges in its treatment [56].

A total of 27 *Penicillium* isolates were identified as seven species belonging to three distinct sections (*Furcatum*, *Aspergilloides*, and *Penicillium*). The dominant species were *P. chrysogenum* (37.0%) and *P. citrinum* (22.2%). These species are the potential producers of CTN.

### 3.3. Mycotoxin-Producing Abilities of Aspergillus and Penicillium Isolates

Aflatoxins and ochratoxins are among the most important mycotoxins, and their producers are predominantly found within the genus *Aspergillus*, particularly in *Aspergillus* section *Flavi* [20]. However, *Aspergillus* section *Nigri* species, such as *A. niger* and *A. welwitschiae*, produce ochratoxins and fumonisin [15]. *Penicillium* is a major PAT- and CTN-producing genus [57,58]. Therefore, 117 isolates (90 *Aspergillus* and 27 *Penicillium* strains) were analyzed for their ability to produce 11 mycotoxins using UPLC–MS/MS.

Eighteen strains produced mycotoxins (Table 3). Approximately 17.6% of the isolates could produce B-type aflatoxins (specifically aflatoxins B_1_ and B_2_; Figure S1), which were isolated from three samples: *Angelicae dahuricae* radix 03-3A and *Nelumbinis* semen 26–3A and 26–5B. *Nelumbinis* semen, commonly known as the lotus seed or lotus seed kernel, is the dried mature seed of *Nelumbo nucifera* and is a commonly used edible substance with important medicinal and edible functions. This herb exhibits various health benefits, including spleen strengthening for diarrhea prevention, kidney nourishment and essence preservation, leukorrhea alleviation, and heart soothing to promote tranquility [59]. *Nelumbinis* semen is rich in protein and is predominantly produced in the warm and humid regions of southern China. It is highly vulnerable to fungal infections, posing considerable risks to its quality [60].

Aflatoxin-producing fungi in *Nelumbinis* semen samples produced in Zhejiang Province were successfully isolated. These findings highlight the potential for aflatoxin contamination of improperly-managed *Nelumbinis* semen. Some studies have indicated that *Nelumbinis* semen is highly likely contaminated with aflatoxins [61,62]. To address this issue, the 2020 version of the Chinese Pharmacopoeia lists 24 varieties of medicinal substances, including *Nelumbinis* semen, as potentially containing aflatoxins, with a permissible limit of 10 µg·kg^−1^. The regulatory measures are designed to guarantee the safety and quality of medicinal materials by setting permissible limits for aflatoxin contamination, thereby safeguarding consumers from potential health risks associated with the ingestion of contaminated products.

None of the *A. flavus* isolates in this study produced G-type aflatoxins. *Aspergillus flavus* is well-known for its ability to produce aflatoxins, particularly B-type aflatoxins (AFB_1_ and AFB_2_). This is consistent with the study by Adelusi et al. [63], who reported that *A. flavus* strains isolated from smallholder dairy cattle feeds in South Africa predominantly produced B-type aflatoxins, with no detection of G-type aflatoxins. This selective production is likely due to genetic differences in the aflatoxin biosynthesis gene cluster, where certain genes required for G-type aflatoxin production may be absent or non-functional in these strains.

Of the 33 strains of *A. niger*, 10 were FB_2_ producers (Figure S3), isolated from *Atractylodis macrocephalae* rhizoma 06–2A; *Acanthopanacis senticosi* radix et rhizoma *seu caulis* 01-3A; *Salviae miltiorrhizae* radix et rhizoma 37–1B and 37–3C; *Rhodiolae crenulatae* radix et rhizoma 35-4A; *Rosae laevigatae* fructus 36–1B; *Dioscoreae* rhizoma 13–2D; *Crataegi* fructus 10–2A; *Leonuri* herba 22–3B; and *Polygonati rodorati* rhizoma 32–1B. One *A. niger* isolate (from *Rhodiolae crenulatae* radix et rhizoma 35–4A) also produced OTA (Figure S2). Only *A. niger* strains isolated from *Alismatis* rhizoma 02–2A and *Gardeniae* fructus 17–1B could produce OTA (Figure S2).

*Aspergillus niger* is common in tropical and subtropical food sources [17], including edible and medicinal substances. The incidence of strains within this species that produce FB_2_ is high. Our observation that a few *A. niger* strains produced ochratoxin A (OTA) aligns with findings from other studies. *Aspergillus niger* is recognized as a potential producer of OTA, although not all strains exhibit this capability. Antonia et al. [64] demonstrated that the presence of OTA biosynthetic genes (e.g., *ota1*, *ota2*, *ota3*, *ota4*, and *ota5*) was crucial for OTA production. In their study, OTA-nonproducing strains of *A. niger* were found to lack these genes, indicating that the genetic potential for OTA production is a key determinant. The current results suggest that the *A. niger* strains producing OTA possessed the necessary biosynthetic genes, whereas the non-producing strains may lack them. In contrast, none of the seven strains of *A. welwitschiae* analyzed in the current study were found to produce either OTA or FB_2_. This indicates that the *A. welwitschiae* strains isolated from edible and medicinal substances in the current study do not possess mycotoxin-producing capabilities. These findings elucidate the mycotoxin-producing potential of *Aspergillus* species in medicinal and edible substances, emphasizing the importance of genetic factors in determining mycotoxin production.

CTN with renal toxicity was detected in three strains of *P. citrinum* (*Lablab* semen *album* 21–2A and *Nelumbinis* semen 26–1C and 26–5D; Figure S4). *Penicillium citrinum* can colonize almost all substrates, including grains, fruits, vegetables, and tea [65,66]. Despite the low isolation rate of *P. citrinum* from edible and medicinal substances, all identified strains produced CTN. Furthermore, we not only isolated CTN-producing strains from *Nelumbinis* semen but also found aflatoxin-producing strains, indicating that *Nelumbinis* semen is at risk of contamination with mycotoxin mixtures. Mycotoxin mixtures in food products pose a considerable health risk owing to their potential synergistic or additive toxic effects [67].

Mycotoxins, such as aflatoxins, ochratoxins, and fumonisins, are secondary metabolites produced by fungi that contaminate various foods and medicinal substances. For instance, the co-occurrence of aflatoxin B_1_ and ochratoxin A exacerbates liver and kidney damage in animal studies [68]. Furthermore, mycotoxin mixtures complicate risk assessment and regulatory control because current safety guidelines often focus on individual mycotoxins rather than their combined effects. This highlights the need for comprehensive monitoring and stricter regulations to address the risks associated with mycotoxin mixtures in food and medicinal products. However, CTN and FB are not listed in the monitoring directory of food standards or pharmacopoeia. Therefore, some edible and medicinal substances and related products may be contaminated with these mycotoxins because of a lack of official legislation. To ensure food safety and protect consumer interests, extensive surveys of CTN and FB residues in edible and medicinal substances must be conducted to establish a scientific foundation for permissible limits of mycotoxins.

Not all strains produce mycotoxins. The 18 mycotoxigenic strains obtained were distributed across a variety of edible and medicinal substances, including radix et rhizoma, semen, fructus, and flos et folium. Half of these mycotoxigenic strains were isolated from radix et rhizoma samples. Mycotoxins, particularly aflatoxins and fumonisins, are common in herbs containing radix et rhizoma [19]. Mycotoxin production is influenced by mycotoxigenic strains and environmental factors (e.g., host, light, temperature, and water activity) [69,70,71]. The presence of mycotoxin-synthesizing gene clusters in the genomes of these strains is a fundamental cause of mycotoxin production. Environmental factors are transmitted to mycotoxigenic strains through signal transduction. The expression of mycotoxin synthesis gene clusters can be activated or inhibited, thereby affecting mycotoxin synthesis [72]. Therefore, cool (≤20 °C) and dry (average relative humidity <40% at 20 °C) storage of the above edible and medicinal substances is recommended (Chinese Pharmacopeia Commission [27]; United States Pharmacopeia Commission [73]). Producers should ensure optimal storage conditions, including temperature, humidity, and ventilation, to minimize the proliferation of potentially mycotoxigenic fungi [74]. Regular monitoring of these conditions is essential to determine the appropriate storage duration and mitigate the risk of mycotoxin accumulation. Decisions regarding the storage period and timely sale of products should be based on these monitoring results [75].

### 3.4. Mycotoxin Content in Edible and Medicinal Substances

Comprehensive analysis of 11 mycotoxins in 163 edible and medicinal substances indicated that 8.0% of samples exceeded the detection limits (Table 4; Figure S5). Mycotoxin contamination was identified in *Angelicae dahuricae* radix, *Chrysanthemi* flos, *Glycyrrhizae* radix et rhizoma, *Mori* fructus, *Nelumbinis* semen, and *Raisin tree* semen. The predominant mycotoxins detected were AFB_1_, AFB_2_, OTA, and ZEN. No traces of the seven other mycotoxins, namely AFG_1_, AFG_2_, FB_1_, FB_2_, FB_3_, PAT, or CTN, were found in any of the samples. The levels of mycotoxins found in these samples ranged from 0.07 to 484.30 μg·kg^−1^, with OTA being the most prevalent (detected in 4.3% of samples). AFB_1_ was detected in 2.5% of samples, whereas ZEN and AFB_2_ were detected in 1.8% and 0.6% of samples, respectively.

The legal threshold for AFB_1_ in food products is typically 2–10 μg·kg^−1^, whereas that for ZEN is 50–200 μg·kg^−1^ [76,77]. One batch of *Nelumbinis* semen had 10.75 μg·kg^−1^ AFB_1_ and one batch of *Raisin tree* semen contained 484.30 μg·kg^−1^ ZEN. The concentrations detected in these batches far exceeded these limits, indicating a potential failure in quality control measures. Furthermore, AFB_1_- and AFB_2_-producing *A. flavus* strains were isolated from *Nelumbinis* semen and *Angelicae dahuricae* radix, suggesting a cumulative risk of aflatoxin exposure to these particular edible and medicinal substances. Although fumonitoxin-producing *A. niger* was detected in various edible and medicinal substances, fumonitoxin was not detected. The absence of detectable mycotoxins in some samples, despite the presence of potentially toxigenic fungi, may have been influenced by optimal storage, transport, or processing conditions.

Appropriate storage (e.g., controlled temperature and humidity), efficient transport practices, and effective processing methods (e.g., drying, cleaning, and sterilization) significantly substantially inhibits fungal growth and mycotoxin production [78]. The fungi may have been dormant or may not have been exposed to conditions conducive to toxin production at sampling. Alternatively, the detection methods used may not have been sensitive enough to detect low levels of fumonitoxin. Further studies on the effect of storage and processing conditions on mycotoxin production using these materials will provide valuable insights.

Mycotoxins in edible and medicinal substances pose a direct threat to consumer health and highlight the need for stringent quality control measures during production and storage. Adherence to competent agricultural practices and effective post-harvest handling procedures are imperative for minimizing fungal growth and mycotoxin production. Regulatory bodies should regularly monitor edible and medicinal substances to ensure compliance with safety standards and protect public health. Further research is required to develop more sensitive and specific detection methods for mycotoxins in edible and medicinal substances and to explore the potential for mycotoxin reduction through processing and treatment methods.

## 4. Conclusions

In this study, the levels of fungi and prevalence of mycotoxigenic fungi in 40 different types of popular edible and medicinal substances from China were systematically evaluated. The study indicated low concentrations of fungi in several edible and medicinal substances, suggesting that most samples were within acceptable fungal contamination limits. The fungal flora of edible and medicinal substances was diverse, with *Aspergillus* being the most prevalent genus, followed by *Penicillium*, which was consistent with the results of previous studies. The ability of the prevalent isolates to produce mycotoxins was also assessed, with 18 strains testing positive for the production of five different mycotoxins. Ochratoxin was of particular concern because strains of *A. niger* produce fumonisin B_2_ and OTA. Strains producing the two mycotoxins were obtained from *Nelumbinis* semen and *R. crenulatae* radix et rhizoma, which may increase the risk of mycotoxin residues. Mycotoxin content analysis of edible and medicinal substances indicated high levels of ZEN in *Raisin tree* semen. Aflatoxin B was detected in *Angelicae dahuricae* radix and *Nelumbinis* semen, and aflatoxin-producing *Aspergillus* spp. were isolated from these samples. These findings emphasize the need for stringent quality control measures during the production, processing, and storage of edible and medicinal substances to minimize fungal contamination and mycotoxin production. Regular monitoring and the establishment of limits for mycotoxins in edible and medicinal substances are crucial for ensuring consumer safety. Further research is warranted to develop more sensitive detection methods for mycotoxins and explore potential reduction strategies through processing and treatment. In addition, the establishment and improvement of a mycotoxigenic strain resource bank will support research on the biological characteristics, production mechanisms, detection methodologies, and control strategies of mycotoxin-producing fungi.

## Figures and Tables

**Table 1:** Forty different species of edible and medicinal substances.

Scientific Name *	Type of Edible and Medicinal Substances	Number of Samples	Sample Name	Producing Regions
*Acanthopanacis senticosi* radix et rhizoma *seu caulis*	radix et rhizoma	4	01-1; 01-2; 01-3; 01-4	Heilongjiang
*Alismatis* rhizoma	radix et rhizoma	3	02-1; 02-2; 02-3	Sichuan
*Angelicae dahuricae* radix	radix et rhizoma	3	03-1; 03-2; 03-3	Sichuan
*Angelicae sinensis* radix	radix et rhizoma	4	04-1; 04-2; 04-3; 04-4	Gansu
*Asini corii colla*	animal	3	05-1; 05-2; 05-3	Shandong
*Atractylodis macrocephalae* rhizoma	radix et rhizoma	5	06-1; 06-3; 06-4; 06-5	Zhejiang
			06-2	Sichuan
*Auricularia cornea*	edible fungi	5	07-1; 07-5	Jiangsu
			07-2; 07-3; 07-4	Zhejiang
*Cassiae* semen	semen	5	08-1	Hebei
			08-2; 08-3; 08-4; 08-5	Anhui
*Chrysanthemi* flos	flos et folium	5	09-1; 09-2; 09-3; 09-4; 09-5	Zhejiang
*Crataegi* fructus	fructus	8	10-1; 10-2; 10-3; 10-4; 10-5; 10-6; 10-7;10-8	Shandong
*Cuscutae* semen	semen	4	11-1; 11-2; 11-3; 11-4	Inner Mongolia
*Dictyophora inausiata*	edible fungi	6	12-1; 12-2; 12-3; 12-4; 12-5; 12-6	Zhejiang
*Dioscoreae* rhizoma	radix et rhizoma	3	13-1; 13-2; 13-3	Henan
*Euryales* semen	semen	6	14-1; 14-3; 14-4; 14-5; 14-6	Jiangxi
			14-2	Guangdong
*Flammulina velutipes*	edible fungi	4	15-1; 15-2; 15-3; 15-4	Zhejiang
*Ganoderma lingzhi*	edible fungi	4	16-1; 16-3	Shaanxi
			16-2	Zhejiang
			16-4	Sichuan
*Gardeniae* fructus	fructus	3	17-1	Zhejiang
			17-2; 17-3	Jiangxi
*Ginseng* radix et rhizoma	radix et rhizoma	5	18-1; 18-2; 18-3; 18-4; 18-5	Jilin
*Glycyrrhizae* radix et rhizoma	radix et rhizoma	5	19-1; 19-2; 19-3; 19-4; 19-5	Inner Mongolia
*Juglandis* semen	semen	3	20-1	Yunnan
			20-2; 20-3	Hebei
*Lablab* semen *album*	semen	4	21-1; 21-2; 21-3; 21-4	Yunnan
*Leonuri herba*	flos et folium	4	22-1; 22-4	Guangdong
			22-2	Hubei
			22-3	Hebei
*Mori* folium	flos et folium	8	23-1; 23-2; 23-3; 23-6; 23-7; 23-8	Guangdong
			23-4; 23-5	Henan
*Mori* fructus	fructus	4	24-1	Guangdong
			24-2; 24-3	Zhejiang
			24-4	Guangxi
*Mume* fructus	fructus	3	25-1; 25-2; 25-3	Sichuan
*Nelumbinis* semen	semen	5	26-1; 26-2; 26-3; 26-4; 26-5	Zhejiang
*Ophiopogonis* radix	radix et rhizoma	3	27-1; 27-2; 27-3	Sichuan
*Ostreae concha*	animal	3	28-1	Guangdong
			28-2; 28-3	Shandong
*Panacis quinquefolii* radix	radix et rhizoma	3	29-1; 29-2	Liaoning
			29-3	Beijing
*Platycodonis* radix	radix et rhizoma	3	30-1; 30-2	Shaanxi
			30-3	Inner Mongolia
*Polygonati* rhizoma	radix et rhizoma	4	31-1; 31-2; 31-3; 31-4	Zhejiang
*Polygonati rodorati* rhizoma	radix et rhizoma	4	32-1; 32-2; 32-3; 32-4	Hunan
*Puerariae lobatae* radix	radix et rhizoma	3	33-1; 33-2; 33-3	Anhui
*Raisin tree* semen	semen	3	34-1	Zhejiang
			34-2; 34-3	Shaanxi
*Rhodiolae crenulatae* radix et rhizoma	radix et rhizoma	4	35-1; 35-2; 35-3; 35-4	Tibet
*Rosae laevigatae* fructus	fructus	3	36-1	Anhui
			36-2; 36-3	Jiangxi
*Salviae miltiorrhizae* radix et rhizoma	radix et rhizoma	4	37-1; 37-2; 37-3; 37-4	Shandong
*Schisandrae chinensis* fructus	fructus	3	38-1; 38-2; 38-3	Jilin
*Scrophulariae* radix	radix et rhizoma	4	39-1; 39-2; 39-3	Hebei
			39-4	Zhejiang
*Sennae* folium	flos et folium	3	40-1; 40-2; 40-3	Yunnan

* Common names in this column are in italics.

**Table 2:** Fungal load in six types of edible and medicinal substances from China.

Type of Edible and Medicinal Substance	Number of Samples	Log TYMC	Mean of Log TYMC	Median of Log TYMC
Animal	6	0.50~4.00	2.18	2.15
Edible fungi	19	0.50~4.28	2.71	3.00
Flos et folium	20	1.85~5.28	2.98	2.83
Fructus	24	0.50~3.78	1.58	1.30
Radix et rhizoma	64	0.50~4.80	2.21	2.21
Semen	30	0.50~4.08	2.40	2.45

Log TYMC: log total combined yeast and mold count.

**Table 3:** Isolated *Aspergillus* and *Penicillium* strains and mycotoxigenic strains.

	Number of Isolates	Number of Mycotoxigenic Fungi			
		Aflatoxins B_1_ and B_2_	Ochratoxin A	Fumonisin B_2_	Citrinin
*Aspergillus amoenus*	1	-	-	-	-
*Aspergillus chevalieri*	3	-	-	-	-
*Aspergillus creber*	1	-	-	-	-
*Aspergillus egyptiacus*	1	-	-	-	-
*Aspergillus flavus*	17	3	-	-	-
*Aspergillus fumigatus*	2	-	-	-	-
*Aspergillus insuetus*	1	-	-	-	-
*Aspergillus luchuensis*	3	-	-	-	-
*Aspergillus montevidensis*	3	-	-	-	-
*Aspergillus niger*	33	-	3 ^a^	10	-
*Aspergillus ruber*	2	-	-	-	-
*Aspergillus sydowii*	5	-	-	-	-
*Aspergillus tabacinus*	3	-	-	-	-
*Aspergillus tamarii*	2	-	-	-	-
*Aspergillus terreus*	1	-	-	-	-
*Aspergillus tubingensis*	5	-	-	-	-
*Aspergillus welwitschiae*	7	-	-	-	-
*Penicillium glabrum*	1	-	-	-	-
*Penicillium chrysogenum*	10	-	-	-	-
*Penicillium citrinum*	6	-	-	-	3
*Penicillium crustosum*	2	-	-	-	-
*Penicillium oxalicum*	1	-	-	-	-
*Penicillium polonicum*	2	-	-	-	-
*Penicillium solitum*	1	-	-	-	-
*Penicillium sumatrense*	1	-	-	-	-
unclassified *Penicillium*	3	-	-	-	-
Total	117	3	3 ^a^	10	3

- Number of mycotoxigenic fungal isolates was zero. ^a^; one of the three *Aspergillus niger* isolates was an ochratoxin A and fumonisin B_2_ producer. All isolates were negative for six mycotoxins (aflatoxins, G_1_ and G_2_; fumonisins, B_1_ and B_3_; patulin; and zearalenone).

**Table 4:** Mycotoxin content of edible and medicinal substances.

	Number of Sample	Mycotoxin Content (μg·kg^−1^)			
		Aflatoxins B_1_	Aflatoxins B_2_	Ochratoxin A	Zearalenone
*Angelicae dahuricae* radix	03–1	0.16 ± 0.00	-	-	-
	03–2	1.35 ± 0.01	0.07 ± 0.01	-	-
*Chrysanthemi* flos	09–1	0.76 ± 0.01	-	1.29 ± 0.01	-
	09–3	-	-	3.48 ± 0.02	-
*Glycyrrhizae* radix et rhizoma	19–1	-	-	2.54 ± 0.07	-
	19–4	-	-	2.84 ± 0.01	-
	19–5	-	-	8.82 ± 0.05	-
*Mori* fructus	24–2	-	-	3.33 ± 0.02	-
	24–4	-	-	0.98 ± 0.02	-
*Nelumbinis* semen	26–1	10.75 ± 0.16 ^a^	1.31 ± 0.04	-	-
*Raisin tree* semen	34–1	-	-	-	484.30 ± 6.18 ^b^
	34–2	-	-	-	38.23 ± 2.48
	34–3	-	-	-	35.05 ± 10.42

Note: ^a^ is excessive (according to Pharmacopoeia of the People’s Republic of China, 2020, AFB_1_ may not exceed 5.0 μg·kg^−1^ in *Nelumbinis* semen); ^b^ is excessive (according to GB 2761-2017 [76], ZEN may not exceed 60 μg·kg^−1^ in wheat and corn); AF, aflatoxin; ZEN, zearalenone.

## Data Availability

The original contributions presented in the study are included in the article/Supplementary Materials. Further inquiries can be directed to the corresponding authors.

## Supplementary Materials

The following supporting information can be downloaded at: https://www.mdpi.com/article/10.3390/jof11030212/s1, Table S1. Optimal multiple reaction monitoring transitions, declustering potential, dwell time (ms), collision energies of mass analysis performed using electrospray ionization; Table S2. Linearity equations, correlation coefficients, limits of detection (LODs), and limits of quantification (LOQs) of the method (LODs and LOQs were measured using standard solutions); Table S3. The recoveries (Rs) and relative standard deviations (RSDs) for six different edible and medicinal substances with mycotoxins; Table S4. Isolates from China examined in this study. Figure S1. Ability of fungi to produce aflatoxins. (a–d) Standard solution: AFB_1_; AFB_2_; AFG_1_; AFG_2_ (e,f) *Aspergillus flavus*; Figure S2. Ability of fungi to produce ochratoxin A. (a) Standard solution: OTA, (b) *Aspergillus niger*; Figure S3. Ability of fungi to produce fumonisin. (a–c) Standard solution: FB_1_; FB_2_; FB_3_, (d) *Aspergillus niger*; Figure S4. Ability of fungi to produce citrinin. (a) Standard solution: citrinin, (b) *Penicillium citrinum*; Figure S5. HPLC–FLD chromatograms for (a) AFB_1_, AFB_2_, AFG_1_, and AFG_2_ standard (AFB_1_, AFG_1_ = 10 ng·mL^−1^; AFB_2_, AFG_2_ = 3 ng·mL^−1^); (b) AFB_1_-, AFB_2_-positive sample (*Angelicae dahuricae* radix 03-2); (c) OTA standard (OTA = 10 ng·mL^−1^); (d) OTA-positive sample (*Chrysanthemi* flos 09-1); (e) ZEN standard (ZEN = 200 ng·mL^−1^); and (f) ZEN-positive sample (*Raisin tree* semen 34-1).

## Abbreviations

The following abbreviations are used in this manuscript:

AF	aflatoxin
CTN	citrinin
CYA	Czapek yeast extract agar
F	fumonisin
LOD	limit of detection
LOQ	limit of quantification
MEA	malt extract agar
OTA	ochratoxin A
PAT	patulin
TYMC	total combined yeast and mold count
UPLC–MS/MS	ultra-performance liquid chromatography coupled with tandem mass spectrometry
ZEN	zearalenone
