# Supplementary material for: Occurrence and Diversity of Fungi and Their Mycotoxin Production in Common Edible and Medicinal Substances from China
Source: J Fungi (Basel). 2025 Mar 10;11(3):212. doi: 10.3390/jof11030212 (PMC11943191; doi:10.3390/jof11030212)
Supplement: Supplementary file 1 [file jof-11-00212-s001.zip › jof-3438181_Supplementary_Materials.pdf]

Journal of *Fungi*

MDPI

Supplementary Materials

Occurrence and Diversity of Fungi and Their Mycotoxin Production in Common Edible and Medicinal Substances from China

Table S1. Optimal multiple reaction monitoring transitions, dwell time (ms), declustering potential (DP), and collision energy (CE) of mass analysis performed using electrospray ionization

Compound Name	Species	Precursor ion	Product ion	Dwell time (ms)	DP (V)	CE (V)
Aflatoxin B_1 (AFB_1)	[M+H]+	313.2	241.2<br>285.1	10.0	153	51<br>33
Aflatoxin B_2 (AFB_2)	[M+H]+	315.2	259.1<br>287.2	10.0	150	40<br>37
Aflatoxin G_1 (AFG_1)	[M+H]+	329.0	243.2<br>311.1	10.0	142	38.5<br>31
Aflatoxin G_2 (AFG_2)	[M+H]+	331.1	245.2<br>189.1	10.0	121	41.6<br>55.5
Ochratoxin A (OTA)	[M-H]-	402.1	358.1<br>211.1	10.0	-60	-29<br>-40
Fumonisin B_1 (FB_1)	[M+H]+	722.0	352.3<br>334.3	10.0	148	50<br>55
Fumonisin B_2 (FB_2)	[M+H]+	706.2	336.4<br>688.4	10.0	180	51<br>40
Fumonisin B_3 (FB_3)	[M+H]+	706.3	336.4<br>688.5	10.0	164	50<br>42
Zearalenone (ZEN)	[M-H]-	317.2	131.2<br>175.1	10.0	-140	-38<br>-32
Patulin (PAT)	[M-H]-	153.0	65.1<br>109.0	10.0	-50.0	-17<br>-9
Citrinin (CTN)	[M+MeOH-H]-	281.1	249<br>205.1	10.0	-50.0	-23<br>-32

Table S2. Linearity equations, correlation coefficients (R^2), limits of detection (LOD), and limits of quantification (LOQ) of the method (LODs and LOQs were measured using standard solutions)

Abbreviation	Linear range (μg·kg^{-1})	Linearity equation	R^2	LOD (μg·kg^{-1})	LOQ (μg·kg^{-1})
AFB_1	0.1–20	Y = 160396 X - 14440.4	0.9994	0.03	0.1
AFB_2	0.03–6	Y = 416800 X - 12417.6	0.9994	0.03	0.1
AFG_1	0.1–20	Y = 54332.3 X - 6854.73	0.9992	0.03	0.1
AFG_2	0.03–6	Y = 184644 X - 7372.07	0.9992	0.03	0.1
OTA	1–50	Y = 7653.75 X + 2012.50	0.9999	0.3	1
ZEN	10–500	Y = 21427.47 X - 6914.79	0.9998	5	17

Table S3. The recoveries (R) and relative standard deviations (RSD) for six diffierent edible and medicinal substances with mycotoxins

Mycotoxin	Spiked level (μg·kg^{-1})	*Chrysanthemi flos*		*Angelicae dahuricae radix*		*Nelumbinis semen*		*Mori fructus*		*Glycyrrhizae radix et rhizoma*		Raisin tree semen	
		R (%)	RSD (%)	R (%)	RSD (%)	R (%)	RSD (%)	R (%)	RSD (%)	R (%)	RSD (%)	R (%)	RSD (%)
AFB_1	0.1	89.50	1.46	87.30	3.07	86.90	1.98	-	-	-	-	-	-
	0.5	94.40	1.44	95.30	0.87	96.70	2.95	-	-	-	-	-	-
	1	90.50	0.37	94.80	1.20	96.00	0.18	-	-	-	-	-	-
AFB_2	0.03	90.00	5.34	85.70	4.68	91.10	5.38	-	-	-	-	-	-
	0.15	92.70	1.23	106.70	0.26	98.00	5.16	-	-	-	-	-	-
	0.3	96.00	0.58	106.40	0.32	98.00	0.26	-	-	-	-	-	-
AFG_1	0.1	80.90	4.91	85.90	4.46	79.90	5.02	-	-	-	-	-	-
	0.5	89.00	2.00	97.00	0.84	89.30	2.55	-	-	-	-	-	-
	1	93.40	0.43	97.60	1.56	89.00	0.21	-	-	-	-	-	-
AFG_2	0.03	80.90	5.22	89.90	3.32	85.60	3.18	-	-	-	-	-	-
	0.15	89.80	1.94	105.10	0.93	95.10	0.53	-	-	-	-	-	-
	0.3	98.70	0.35	106.70	1.12	95.80	0.38	-	-	-	-	-	-
OTA	1	91.60	3.66	-	-	-	-	80.10	5.92	84.90	1.76	-	-
	5	95.80	0.83	-	-	-	-	95.20	0.50	91.00	2.18	-	-
	10	99.60	0.57	-	-	-	-	93.30	0.94	95.00	1.59	-	-
ZEN	10	-	-	-	-	-	-	-	-	-	-	90.90	5.37
	50	-	-	-	-	-	-	-	-	-	-	89.70	3.96
	100	-	-	-	-	-	-	-	-	-	-	101.40	0.87

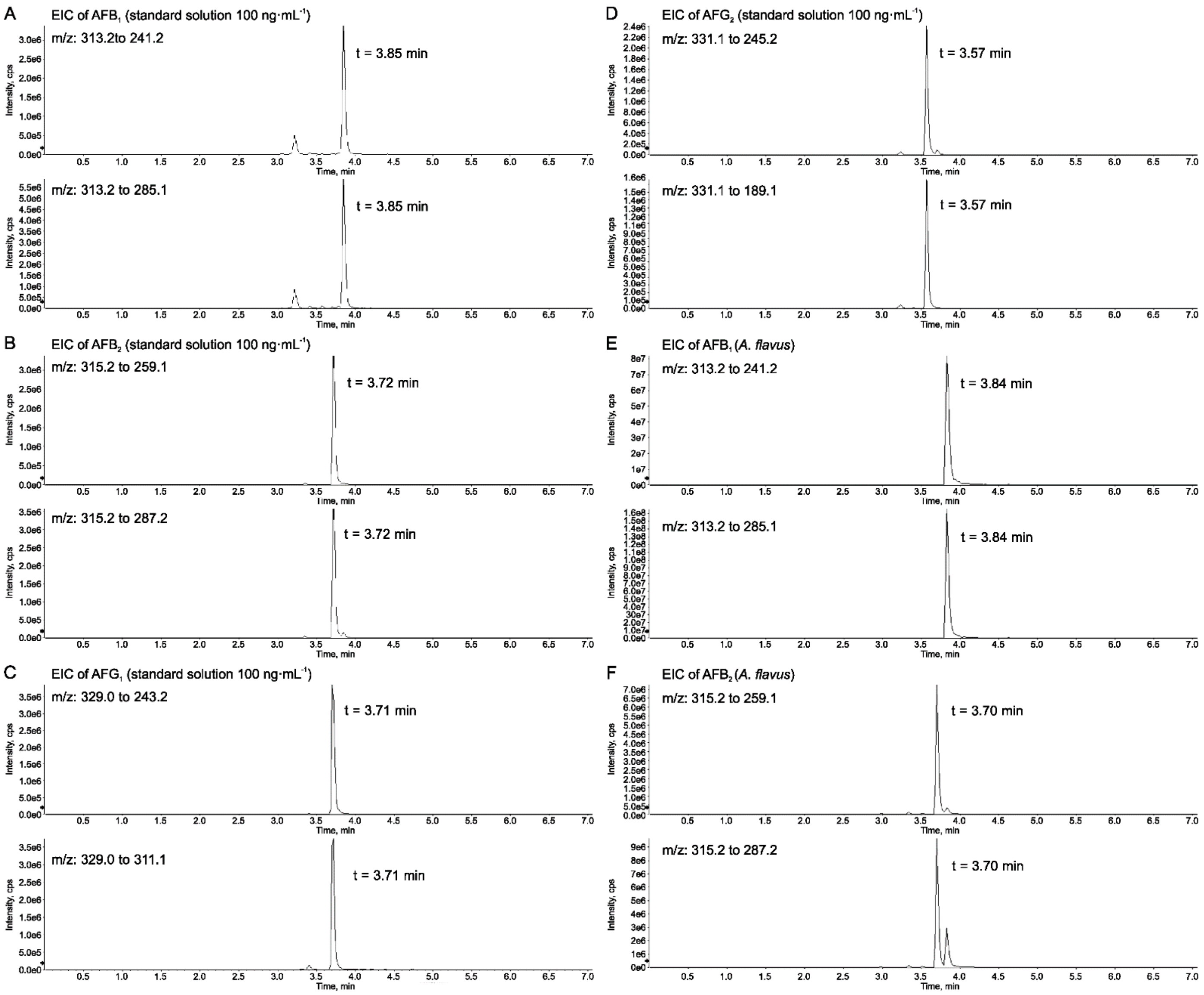

Figure S1. Ability of fungi to produce aflatoxins. (A–D) Standard solutions of AFB_1, AFB_2, AFG_1, and AFG_2; (E,F) *Aspergillus flavus* AF; aflatoxin; EIC, extracted-ion chromatogram

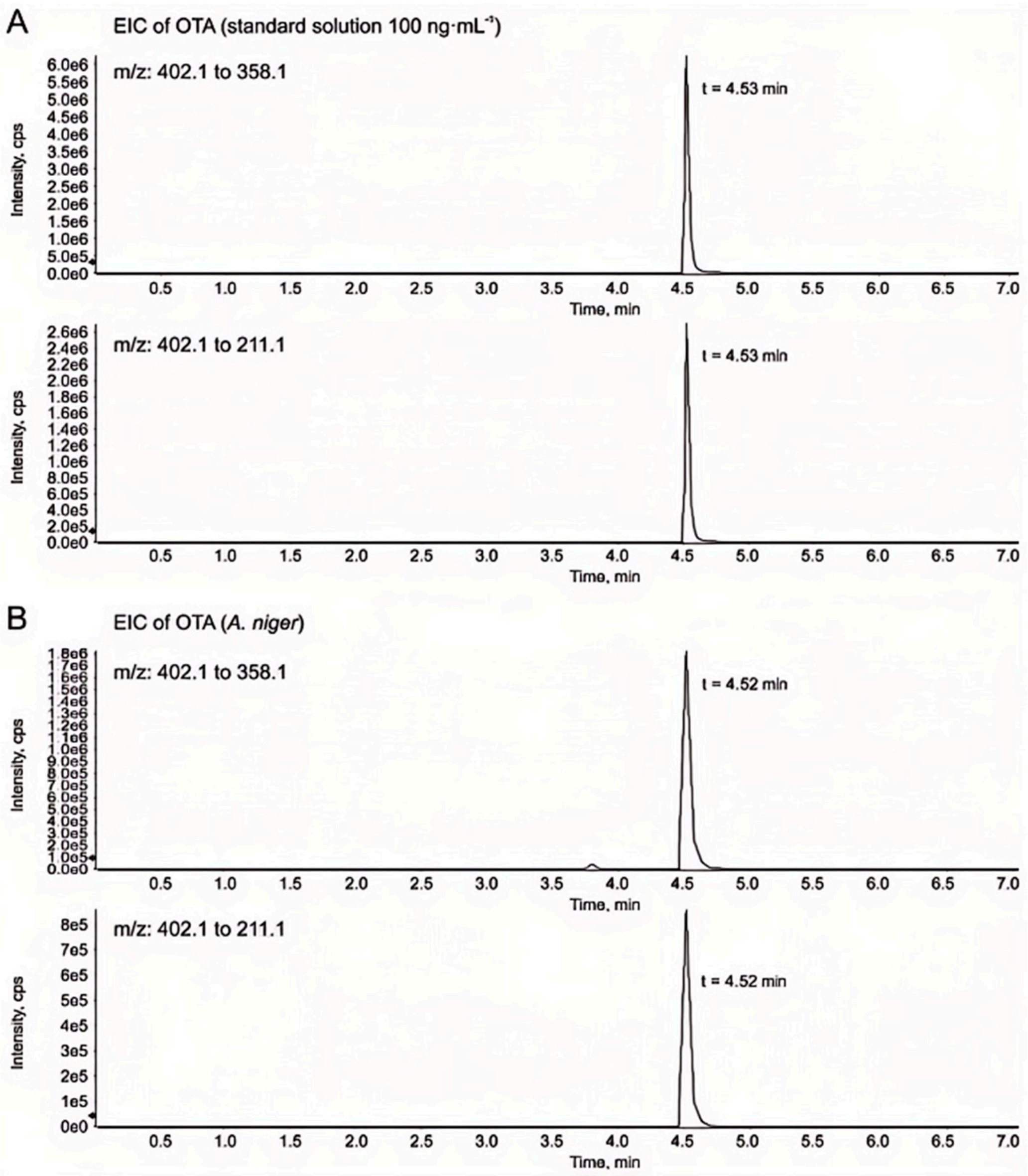

Figure S2. Ability of fungi to produce ochratoxin A. (A) Standard solution, OTA; (B) *Aspergillus niger* EIC, extracted-ion chromatogram; OTA, ochratoxin A

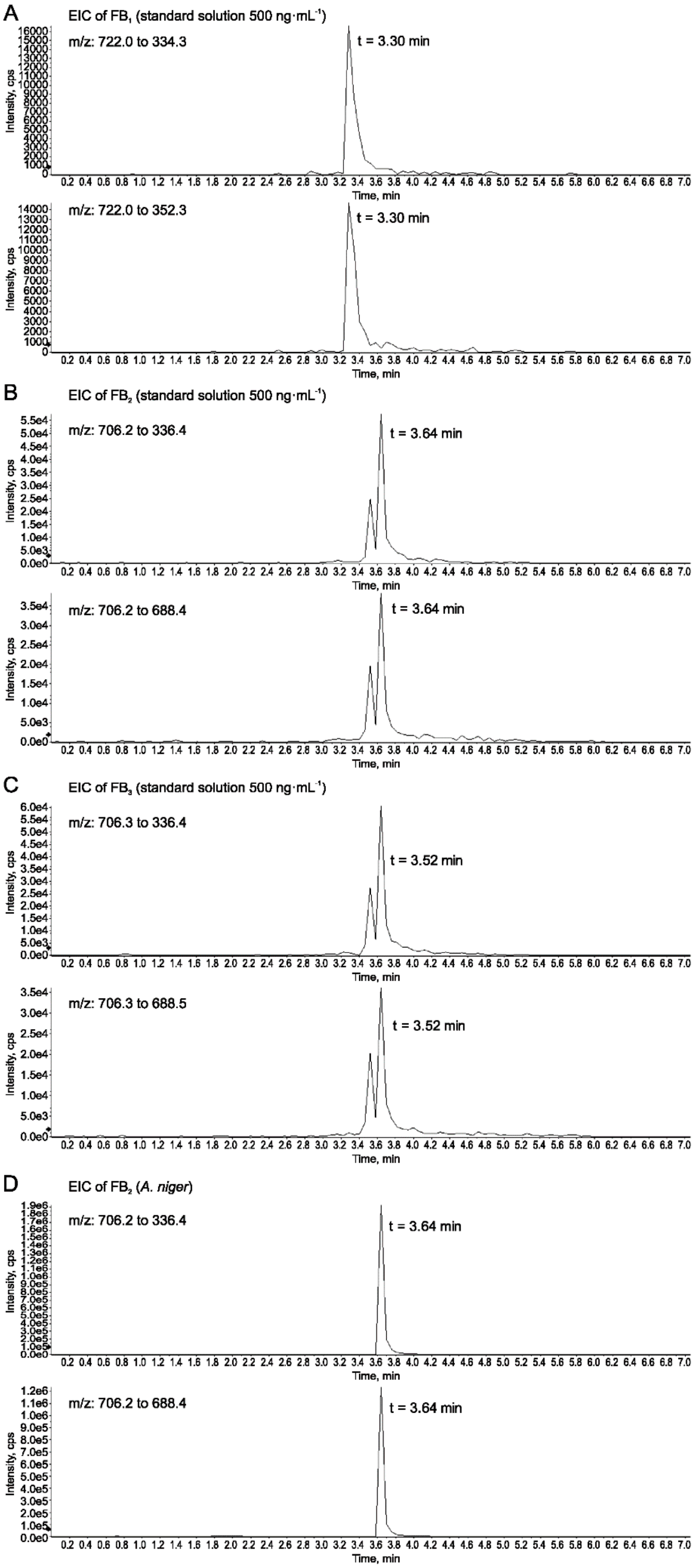

Figure S3. Ability of fungi to produce fumonisin. (A–C) Standard solution: FB_1, FB_2, and FB_3; (D) *Aspergillus niger* EIC, extracted ion chromatogram; FB, fumonisin

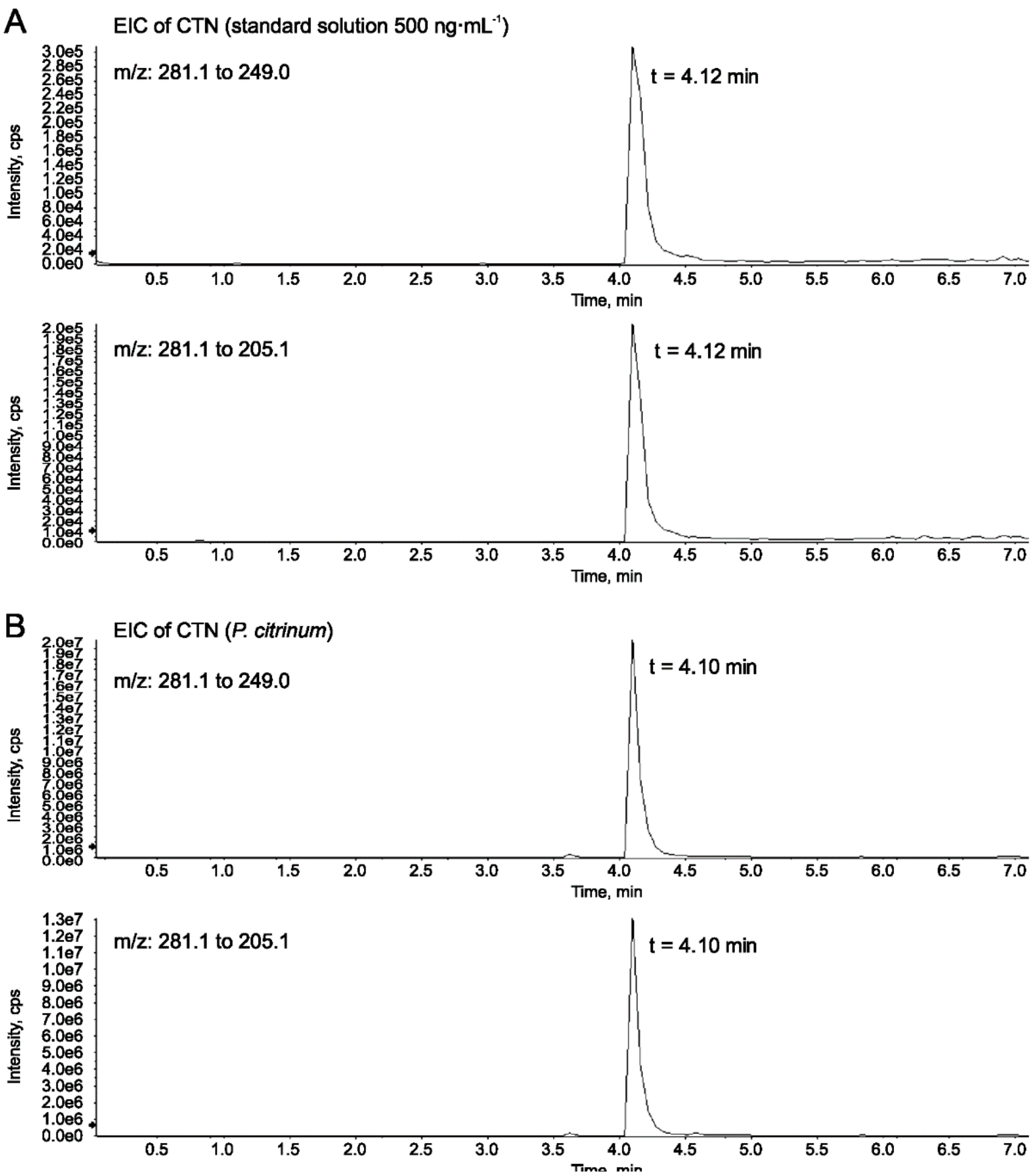

Figure S4. Ability of fungi to produce citrinin. (A) Standard solution: citrinin; (B) *Penicillium citrinum* EIC, extracted-ion chromatogram; CTN, citrinin

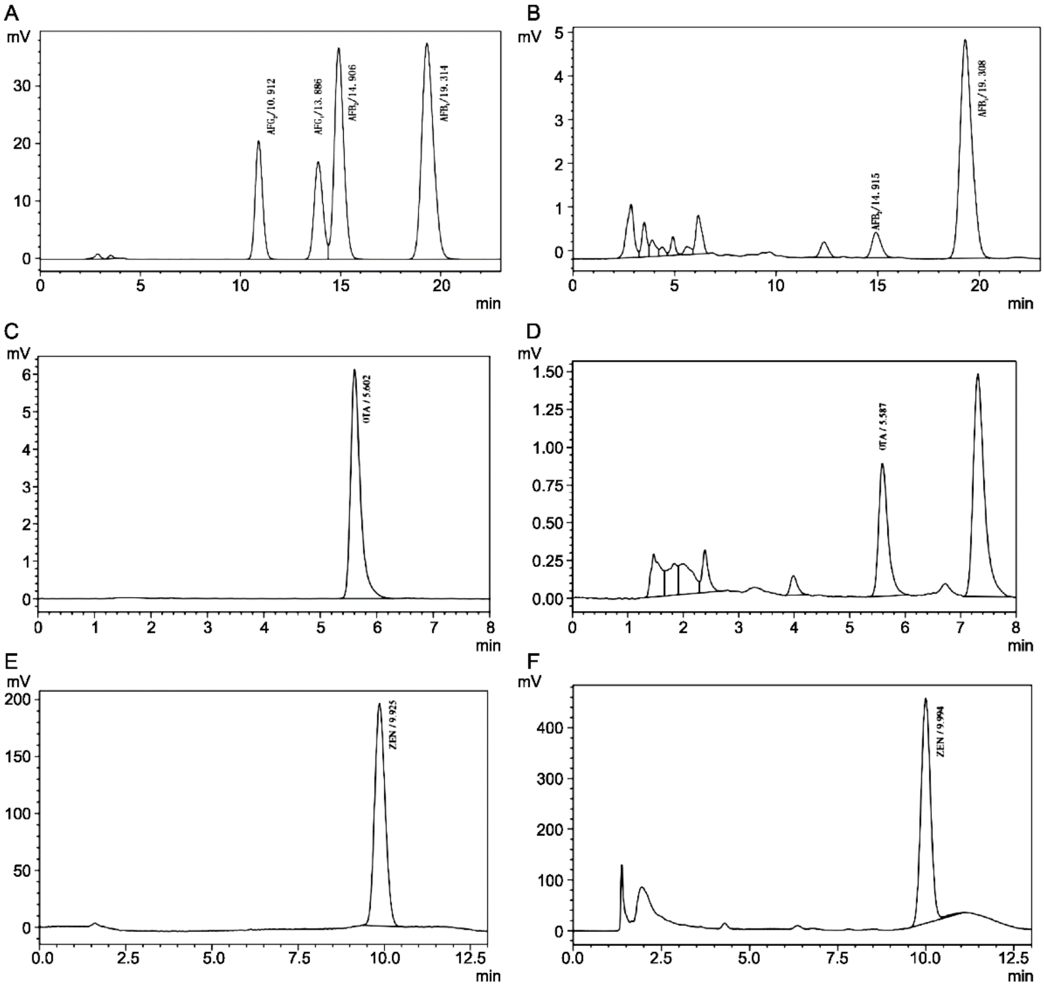

Figure S5. HPLC-FLD chromatograms for (A) AFB_1, AFB_2, AFG_1, and AFG_2 standard (AFB_1, AFG_1 = 10 ng·mL^{-1}; AFB_2, AFG_2 = 3 ng·mL^{-1}); (B) AFB_1-, AFB_2-positive sample (*Angelicae dahuricae* radix 03-2); (C) OTA standard (OTA = 10 ng·mL^{-1}); (D) OTA-positive sample (*Chrysanthemi* flos 09-1); (E) ZEN standard (ZEN = 200 ng·mL^{-1}); and (F) ZEN-positive sample (*Raisin tree* semen 34-1). HPLC-FLD, high-performance liquid chromatography with fluorescence detection; AF, aflatoxin; OTA, ochratoxin; ZEN, zearalenone
